# Liming impacts barley yield over a wide concentration range of soil exchangeable cations

**DOI:** 10.1007/s10705-020-10117-2

**Published:** 2021-04-25

**Authors:** J. E. Holland, P. J. White, J. -N. Thauvin, L. Jordan-Meille, S. M. Haefele, C. L. Thomas, K. W. T. Goulding, S. P. McGrath

**Affiliations:** 1 1 Rhynd Farm Cottages, Leuchars, St Andrews, KY16 0DR UK; 2 James Hutton Institute, Dundee, DD2 5DA UK; 3 Unité Mixte de Recherche 1391 ISPA, Bordeaux Sciences Agro, INRAE, 33140 Villenave d’Ornon, France; 4 Department of Sustainable Agriculture Sciences, Rothamsted Research, Harpenden, Hertfordshire, AL5 2JQ UK

**Keywords:** Soil acidity, Exchangeable cations, Critical concentrations, Soil extraction methods, Long-term experiment

## Abstract

Liming has widespread and significant impacts on soil processes and crop responses. The aim of this study was to describe the relationships between exchangeable cation concentrations in soil and the relative yield of spring barley. The hypothesis was that yield is restricted by the concentration of a single exchangeable cation in the soil. For simplicity, we focused on spring barley which was grown in nine years of a long-term experiment at two sites (Rothamsted and Woburn). Four liming rates were applied and in each year the relative yield (RY) and the concentrations of exchangeable cations were assessed. Liming had highly significant effects on the concentrations of most exchangeable cations, except for Cu and K. There were significant negative relationships (either linear or exponential) between the exchangeable concentrations of Mn, Cd, Cr, Al, Fe, Cu, Co, Zn and Ni in soil and soil pH. The relationships between RY and the concentrations of selected exchangeable cations (Mn, Ca and Al) were described well using log-logistic relationships. For these cations a significant site effect was probably due to fundamental differences in soil properties. At both sites the concentrations of exchangeable soil Al were excessive (> 7.5 mg kg^−1^) and were most likely responsible for reduced barley yields (where RY ≤ 0.5) with soil acidification. At Rothamsted barley yield was non-limited (where RY ≥ 1) at soil exchangeable Mn concentrations (up to 417 mg kg^−1^) greater than previously considered toxic, which requires further evaluation of critical Mn concentrations.

**Supplementary Information:**

The online version of this article (10.1007/s10705-020-10117-2) contains supplementary material, which is available to authorized users.

## Introduction

Acidic soils are a challenge to agriculture and acidification has been identified as one of the most significant degrading processes of soils at the global scale (FAO 2015). Identifying constraints to crop (Holland et al. 2019) and grassland (Stevens and Laughlin 1996) production on acid soils is an important target for current research (Holland et al. 2018). Typically, acidic soils (< pH 4.5) have elevated concentrations of those exchangeable cations (e.g. Al, Mn), which can restrict plant growth. Consequently, much research has been focused upon the effects of acidity on plant growth, and root growth in particular. Major differences exist between crop types and cultivars in their tolerance to acidity (Fageria et al. 2010; Holland et al. 2018) and there are different perceptions within agriculture on the scale and nature of acidity as a problem.

Liming is a common management strategy to ameliorate acidic soils that has multiple long-term effects on soils, crops and biodiversity (Holland et al. 2018). The main impact of liming is an increase in soil pH (Goulding et al. 1989) but, the application of limestone can also change the rate and nature of several soil processes. Liming can increase the Ca and Mg in the soil solution (Bailey 1995; White and Greenwood 2013) and increase the adsorption on clay surfaces of cations such as K, Cu, Co and Zn (Bolan et al. 2003). Liming can also reduce the uptake of potentially harmful cations such as Cd or Zn by crops in contaminated soils (Hooda and Alloway 1996).

Understanding how the concentrations of soil exchangeable cations affect plant growth has been useful for the nutrient management of crops (White and Greenwood 2013). Previous research has employed numerous different soil extraction methods to determine exchangeable cation concentrations in soil (Rayment and Lyons 2011), which are thought to represent the cation concentrations available to plants (Smolders et al. 2009). Researchers have sought to establish critical concentrations of exchangeable cations in soil resulting in crop deficiencies and toxicities (Hazelton and Murphy 2007; Peverill et al. 1999). For many cations, a wide range of soil exchangeable concentrations have been reported as toxic for the same crop (e.g. barley) (Table 1). This is partly a consequence of the use of different soil extraction methods. Basic soil properties such as texture, mineralogy and organic matter influence the concentrations of cations extracted by a given method (Yin et al. 2002). In addition, the dynamic nature of nutrient processes in the soil, such as leaching, plant uptake, and recycling in the soil biota (White 2013), mean that has been difficult to establish critical concentrations of soil exchangeable cations impacting crop production using soil test methods. For example, Carvalho et al. (2015) reported that it is not possible to predict Mn toxicity to crops using current soil test methods. Nevertheless, with current methods it is possible to determine the most likely nutrients/elements for which there might be deficiencies or toxicities.

**Table 1:** Published critical concentrations^a^ of exchangeable cations in the soil (mg kg^−1^) above which toxicity is observed for barley

Cation	Concentration (mg kg^−1^)	Extraction method	Plant response	Reference
Mn	30	0.01 M CaCl_2_	Grain	Slattery and Coventry (1993)
Mn	24–52	0.01 M CaCl_2_	Grain	Dickson et al. (1995)
Cd	30	1 M MgC1_2_	Grain	Dudka et al. (1996)
Cd	60	1 M KCl	Biomass	Wyszkowski and Wyszkowska (2009)
Cr	100–150	–	Biomass	Wyszkowski and Radziemska (2010)
Al	23–24	0.01 M CaCl_2_	Grain	Dolling et al. (1991)
Al	2.5–4.5	0.01 M CaCl_2_	Grain	Anderson and Bell (2019)
Fe	30 ≥ 1000	–	–	Peverill et al. (1999)
Cu	0.3	Amm. oxalate	Grain	Peverill et al. (1999)
Cu	9.0	1 M NH_4_NO_3_	Shoot	Hamels et al. (2014)
Co	30	NA	Shoot	Kapustka et al. (2006)
Zn	60–280	1 M NH_4_NO_3_	Shoot	Hamels et al. (2014)
Ni	28	NA	Shoot	Kapustka et al. (2006)

^a^The concentrations are extractable with the method given for each study

In previous research we tested crop yield effects for lime treatments in a long-term experiment (Holland et al. 2019). This indicated that there were other influences on crop yield, possibly related to cation concentrations. The main aim of this paper was therefore to describe the relationships between exchangeable cation concentrations in soil and the relative yield of spring barley, the most frequently grown crop in the same Long-term Liming Experiment at Rothamsted Research, UK. The hypothesis was that yield is restricted by the concentration of a single exchangeable cation in the soil. In this study the restriction in yield could be due to deficiency or toxicity. Thus the objectives were: (*i*) to describe the relationships between soil pH and soil exchangeable cation concentrations, (*ii*) to describe the relationships between soil exchangeable cation concentrations considered to be toxic or deficient and the relative yield (for all years, n = 9) of spring barley, (*iii*) to evaluate the effect of site (soil type) and the concentration of selected exchangeable cations which might be responsible for the reduced yields of barley in acidic soils.

## Materials and methods

### Experimental site description, experimental design and climate

The Long-term Liming Experiment was undertaken at two sites at Rothamsted Research (Rothamsted and Woburn farms) between 1962 and 1996. The Rothamsted site was in Sawyers field at Rothamsted Research, Harpenden, Hertfordshire, UK (51.8157 N, 0.3752 W). The Woburn site was in Stackyard field (section C) at Woburn Experimental Farm, Husborne Crawley, Bedford, UK (52.0003 N, 0.6149 W), approximately 31 km from Rothamsted. Four rates (main plot treatments) of limestone were applied as ground chalk (CaCO_3_). The liming treatments were control (zero lime), and low (L), medium (M) and high (H) rates of lime. Over the course of the experiment (35 years duration) the total amounts added were 15 and 9 t CaCO3 ha^−1^ for the L treatment, 24.5 and 25.5 for the M treatment and 52.5 and 45.5 for the H treatment for Rothamsted and Woburn respectively. Lime was applied on similar dates at each site and in six separate applications: twice in 1962 and once in 1978, 1981, 1982 and 1986. A detailed description of the experimental sites, including information on the experimental design, the crops grown, and some aspects of the management are provided by Holland et al. (2019). Selected soil properties for the Rothamsted and Woburn sites are given in Table S1. Additional data and descriptions of these soils, including their mineralogy, are available for Rothamsted (Avery and Catt 1995; Tye et al. 2009; Weir et al. 1969) and Woburn (Anon 1977; Catt et al. 1980).

### Soil chemical analysis

Soil exchangeable cation concentrations were determined on selected soil samples from the Rothamsted soil sample archive. Research by Blake et al. (2000) showed that changes in the concentrations of exchangeable cations in the stored samples from the sample archive were minimal. Soil exchangeable chemical Soil exchangeable chemical analyses were undertaken on samples from all liming treatments that were collected in the following years: 1964, 1967, 1974, 1979, 1983 and 1989. Soils were extracted using a 1 M NH_4_NO_3_ solution (Rhoades 1982). Concentrations of exchangeable cations were assayed using inductively coupled plasma optical emission spectrometry (ICP-OES). The detection limit was calculated as 3 × the standard deviation of the blanks and the quantification limit was calculated as 10 × the standard deviation of the blanks. The effective cation exchange capacity (ECEC) was calculated from the sum of the charges of exchangeable cations (Al^3+^, Ca^2+^, Mg^2+^, K^+^ and Na^+^, but excluding H^+^) and expressed as cmol (+) per kg soil using samples from six years (1964, 1967, 1974, 1979, 1983, 1989) from the medium liming treatment. During the long-term liming experiment the soil pH was measured in 1: 2.5 soil: water suspensions using a standard electrode and pH meter. Crop grain yields have been standardised and are reported at 85 percent dry matter. Further details on the field sampling and soil sample analysis are available (Bolton 1977; eRA 2017; Holland et al. 2019).

### Data analysis

Analysis of variance (ANOVA) was used to test the soil exchangeable cation concentrations for main (e.g. lime) treatment effects on spring barley. Data analysis was undertaken in a step-wise manner to investigate the relationships between crop yield for spring barley and the concentrations of exchangeable cations. Spring barley was grown in nine years at both sites during the long-term liming experiment (1962–1996). Because the concentration of exchangeable cations in soil was only measured for one year (1967) when spring barley was grown, it was necessary to estimate the concentrations of exchangeable cations for the other eight years when spring barley was grown (1965, 1966, 1970, 1971, 1972, 1973, 1978, 1985). The concentrations were estimated from the relationships between the concentrations of exchangeable cations in the soil and soil pH which are based equations are given in Table S2 and these data are shown in Fig. 2.

A standard linear and nonlinear regression analysis was used to explore the relationship between the soil pH and the concentrations of exchangeable cations in soil. The basic principle adopted was to use the most simple function available to describe the data, e.g. for pH-Mn a linear equation was used, but for pH-Al an exponential function was selected. Several different functions were tested and the one with the best fit was selected and the relevant metrics (*P* value, R^2^ value and parameter estimates with SE) were calculated accordingly. For Mg and K, there were no clear or obvious relationships with soil pH and these were excluded from further analyses.

The relationship between the grain yield and soil pH were examined using a log-logistic function (four parameter):

1 $$f\left( y \right) = c + \frac{d - c}{{1 + \exp \left( {b\left( {\log \left( x \right) - \log \left( e \right)} \right)} \right)}}$$

where *y* = yield, *x* = soil pH; *b* = slope, *c *= lower limit and *d* = upper limit of yield (Y_ul_); *e* = EC50 (the effective concentration half way (50%) between *c* and *d*). At each experimental site and for each year Y_ul_ was determined independently. Relative yield (RY) was calculated as actual yield (*y*) divided by the Y_ul_ (i.e. *y*/Y_ul_). RY was used instead of actual yield because of the strong seasonal/year differences. The use of RY effectively standardises yield which makes it easier to analyse data from several years together to test for a treatment effect or undertake regression of a specific relationship. The log-logistic function is a sigmoid curve which is commonly selected to describe plant biomass or height and it is informative as each parameter of the function corresponds to an aspect of plant growth (Archontoulis and Miguez 2015). All soil pH, RY and exchangeable cation data were fitted using the ‘drc’ package in R (Ritz et al. 2015). Further statistical analyses were performed in R (R Core Team 2013). Parameter coefficients and the relevant metrics (*P* value, R^2^ value) were calculated accordingly.

The relationship between the concentrations of exchangeable cations in soil and soil pH was used to estimate concentrations for all years except 1967 when measured concentrations were available. Thus, the relationship between concentration of exchangeable cations in soil and relative yield (RY) was evaluated using Eq. 1, except *y* = RY and *x* = concentration of exchangeable cations in soil (mg kg^−1^). All data were checked for the assumptions of normality and transformation was not required. The regression analysis was undertaken for each site and year separately. In addition, all years were tested together for each site using Eq. 1. The standard error for each parameter for Eq. 1 was calculated. Testing for the significance between the sites was determined on the *e* parameter from Eq. 1. The difference was calculated on a 95% confidence interval where either side of the mean was greater than 1.96 × SE. All regression analysis was undertaken with R (R Core Team 2013).

## Results

### Liming and site effects on soil pH and the concentrations of exchangeable cations in the soil

Treatment (site and lime) effects and their interaction were tested on measurements of soil pH and the concentrations of exchangeable cations in the soil for 1967 at each site (Rothamsted and Woburn) (Table 2). Site had a significant (*P* < 0.01) effect on soil pH and on the concentrations of most exchangeable cations except for Al and Cu. There were highly significant (*P* < 0.01) effects of liming on the concentrations of most exchangeable cations, except Cu and K, at both sites. The interaction effects between the treatments were mostly significant (*P* < 0.01), except for Al, Cu, Zn, Mg and K where there was no significant interactions.

**Table 2:** The significance level (*P* value) for the effects of site and lime treatment and their interaction on soil pH and on the concentrations of exchangeable cations (Mn, Ca, Cd, Cr, Al, Fe, Cu, Co, Zn, Ni, Mg, K) in soil for 1967 at Rothamsted and Woburn

Element	Site	Lime	Site × Lime
pH	< 0.001	< 0.001	< 0.001
Mn	< 0.001	< 0.001	< 0.001
Ca	< 0.001	< 0.001	< 0.001
Cd	< 0.001	< 0.001	< 0.001
Cr	< 0.001	< 0.001	< 0.001
Al	0.769	< 0.001	0.663
Fe	0.001	< 0.001	< 0.001
Cu	0.061	0.428	0.062
Co	< 0.001	< 0.001	< 0.001
Zn	0.028	< 0.001	0.023
Ni	0.005	< 0.001	0.006
Mg	< 0.001	< 0.001	0.238
K	< 0.001	0.393	0.754

### The relationship between relative yield (RY) and soil pH

**Fig. 1:**
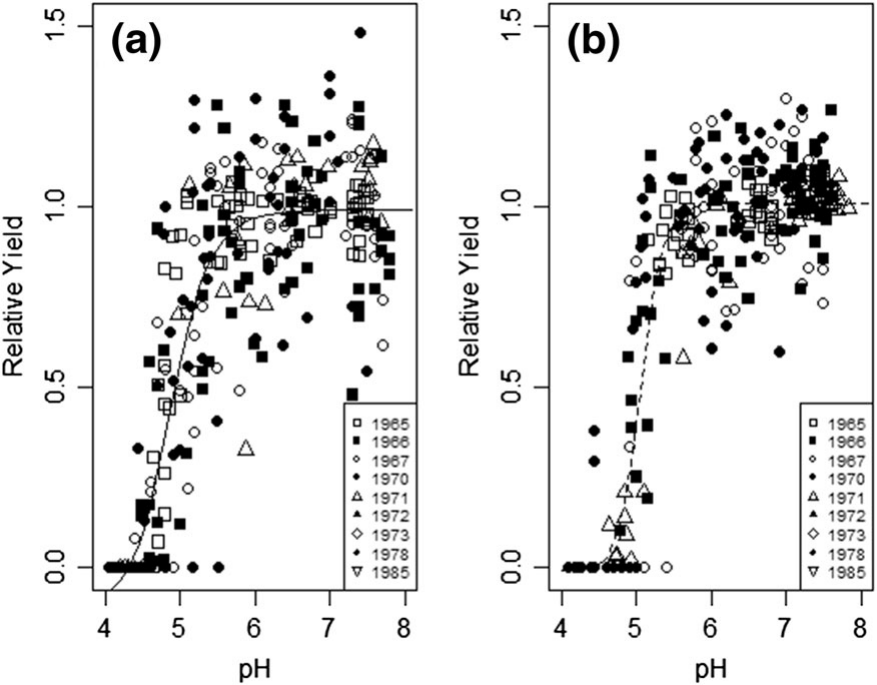
The relationship between relative yield (RY) and soil pH in spring barley crops grown in nine years (1965, 1966, 1967, 1970, 1971, 1972, 1973, 1978, 1985) at **a** Rothamsted and **b** Woburn; parameter coefficients for the regression can be found in Table 3

The relationships between RY and soil pH were evaluated on nine years of spring barley crops at Rothamsted and Woburn (Fig. 1). There was a large range in soil pH at both sites with the minimum soil pH approaching 4 and the maximum pH approaching 8. Both sites had a wide range of RY values from zero (failed crops) to > 1.3 (high yielding crops). The four-parameter log-logistic function (Eq. 1) fitted the relationship between RY and pH for both sites. The Rothamsted data had slightly greater variability than the Woburn data and included more outlier values. Parameter coefficients for the log-logistic relationship between RY and pH are given in Table 3. The EC50 value for Woburn (pH = 5.06) was significantly (*P* < 0.05) greater than the EC50 value at the Rothamsted (pH = 4.88) site.

**Table 3:** Log-logistic (Eq. 1) parameter coefficients for the relationships between soil pH and relative yield (RY) of spring barley crops grown in nine years (1965, 1966, 1967, 1970, 1971, 1972, 1973, 1978, 1985) at Rothamsted and Woburn

Site	B (Slope)	c (Lower Limit)	d (Upper Limit)	e (EC50^a^)
Rothamsted	− 18.6 (3.1)	− 0.10 (0.08)	0.99 (0.02)	4.88 (0.06)
Woburn	− 33.6 (6.4)	− 0.01 (0.05)	1.01 (0.01)	5.06 (0.03)

Standard error of the mean (where n = 288) is given in brackets

^a^The effective concentration, i.e. soil pH at 50% RY

### The relationships between soil pH and the concentrations of exchangeable cations in the soil

**Fig. 2:**
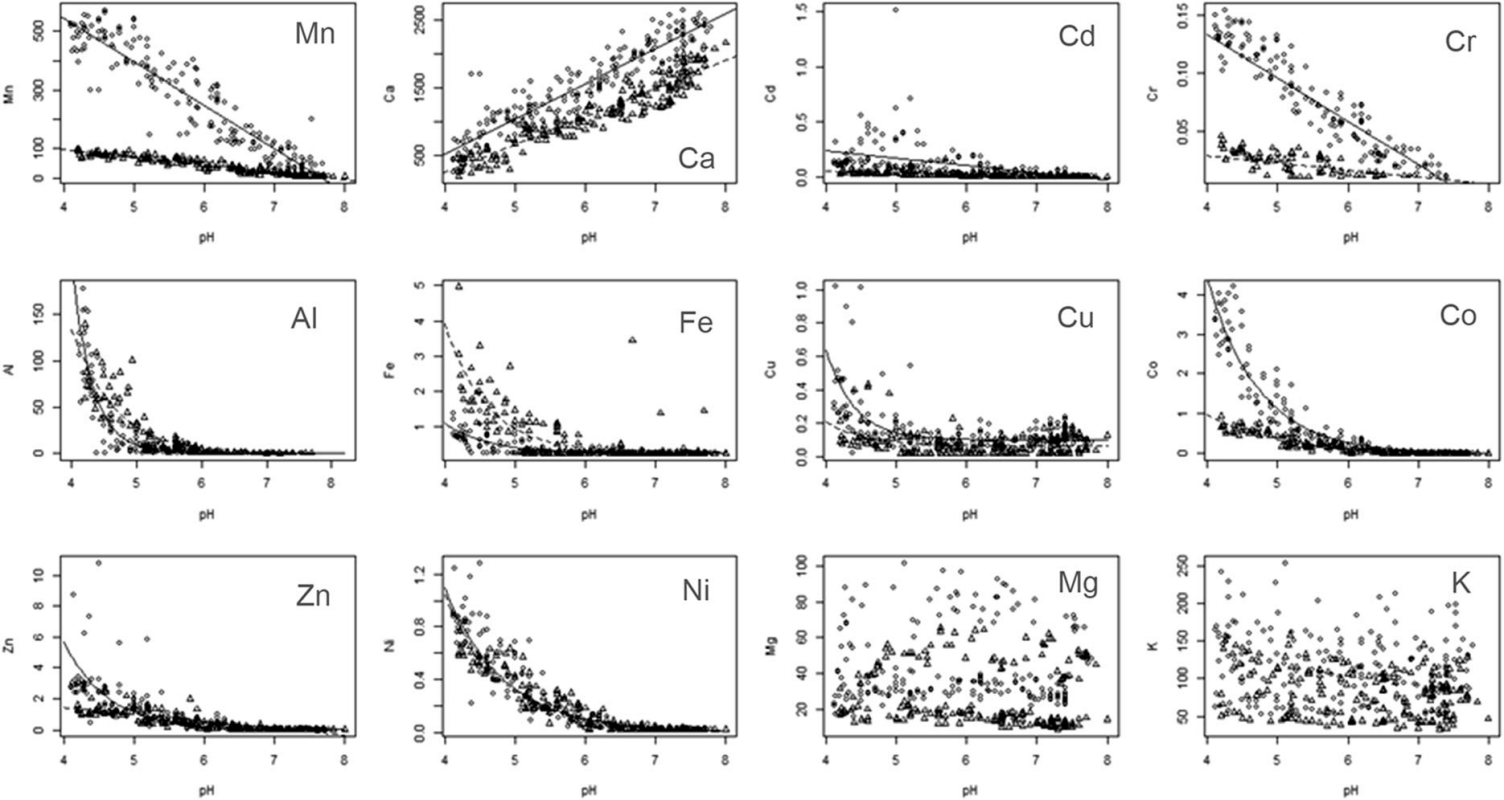
The relationship between the concentrations of the exchangeable cations (Mn, Ca, Cd, Cr, Al, Fe, Cu, Co, Zn, Ni, Mg, K) in the soil (mg kg^−1^) and soil pH (1: 2.5, soil: water) based on measured values from 1964, 1967, 1974, 1979, 1983 and 1989 at Rothamsted (white circle) and Woburn (white triangle)

The relationships between the concentrations of exchangeable cations in the soil and soil pH were described by either: (*i*) linear or (*ii*) exponential functions (Fig. 2). The equations describing these relationships and the associated statistical metrics (*P* value and R^2^) are given in Table S2. There was no relationship between soil pH and Mg or between soil pH and K (Fig. 2) and thus no equation is given in Table S2 for Mg or K. For three exchangeable cations (Mn, Cd and Cr) there was a highly significant (*P* < 0.001) negative linear relationship with soil pH at both sites. In contrast, there was a positive relationship between soil pH and the concentration of Ca. For the concentrations of all exchangeable cations, except Zn, the nature (form) of their relationships with soil pH was the same at both sites. There was a significant difference between the sites in the form of the relationship between soil pH and the concentration of exchangeable Zn. At Woburn there was a linear relationship between soil pH and the concentration of exchangeable Zn, while at Rothamsted there was an exponential relationship (Fig. 2). There were five exchangeable cations (Al, Fe, Cu, Co, Ni) where an exponential function described the relationship with soil pH at both sites (Fig. 2). For Ni there was no significant difference (*P* > 0.05) between the sites in the relationship between soil pH and exchangeable Ni, however there were small differences for the other four cations.

### Correlations between the concentrations of exchangeable cations in the soil

The correlations between the concentrations of exchangeable cations in the soil were calculated (Table S3). Correlation coefficients were mostly very high, but there was some variation in strength (from 0.48 to 1.00) and in direction ranging from positive to negative. There was a negative correlation between the concentration of exchangeable Ca and the concentrations of all the other exchangeable cations. The correlations between all the other exchangeable cations were positive. The weakest correlations were between exchangeable Cu and other exchangeable cations at Woburn. There were some differences in the correlation coefficients between the sites, with the largest differences being between Al, Cu and Zn and other exchangeable cations.

### The relationships between relative yield (RY) and the concentration of exchangeable cations in the soil

**Fig. 3:**
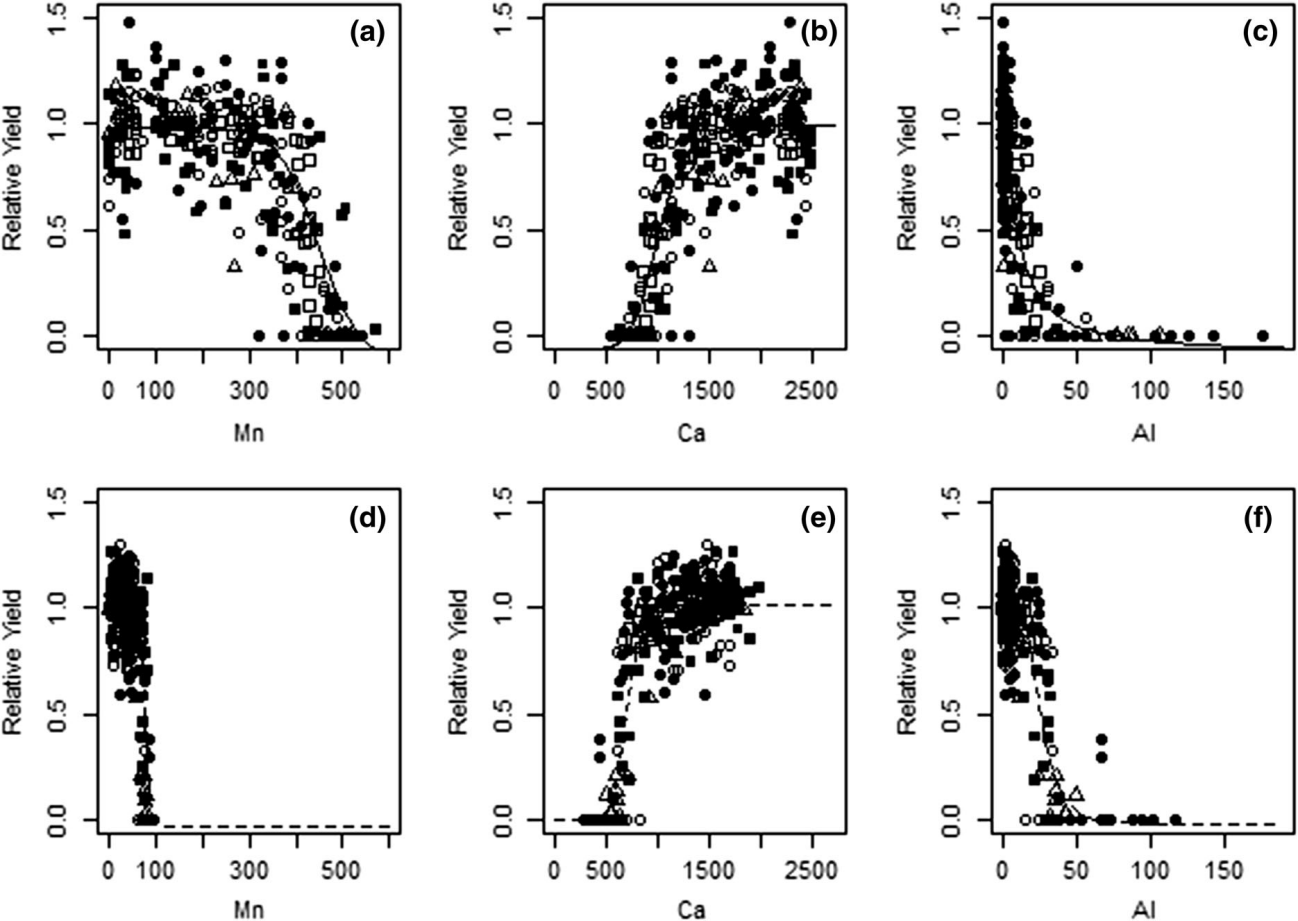
The relationship between relative yield (RY) and concentrations of exchangeable cations (Mn, Ca, Al) in the soil (mg kg^−1^) for spring barley crops in nine years (white square 1965, black filled square 1966, white circle 1967, black filled circle 1970, white triangle 1971, black filled triangle 1972, white rhombus 1973, black filled rhombus 1978, white inverted triangle 1985) at Rothamsted for RY-Mn (**a**), RY-Ca (**b**), RY-Al (**c**) and at Woburn for RY-Mn (**d**), RY-Ca (**e**), RY-Al (**f**). The concentrations in 1967 were measured and the concentrations for all other years were estimated using the equations given in Table S2

The relationships between RY and the concentrations of the exchangeable cations in the soil were only evaluated for three cations (Al, Ca, Mn) for nine years of spring barley crops at Rothamsted and Woburn (Fig. 3). Other cations (Cd, Cr, Fe, Cu, Co, Zn and Ni) had very low exchangeable concentrations (Fig. 2) which were not considered to be toxic. Thus, the relationships between RY and the concentration of these cations was not evaluated. Indeed, comparison between the concentrations (Fig. 2) and published critical concentrations (Table 1) provides strong support that there was no toxicity from Cd, Cr, Fe, Cu, Co, Zn or Ni. Moreover, the concentrations of K and Mg were considered neither deficient nor toxic and thus their relationships with RY were not evaluated. There was a wide range in the concentrations of the exchangeable cations at each site and between sites (Fig. 3). For each site the observed variations in concentrations are due to significant differences between years, but overall, the differences between years were less than the differences between the sites. There was a wide range in the RY, from low yielding crops (RY < 0.3) to high yielding crops (> RY 1.3) (Fig. 3). At both sites there were cases where the RY was zero indicating that the crop failed (Fig. 3). The four-parameter log-logistic function (Eq. 1) was used to describe the relationships between RY and the concentration of exchangeable cations in the soil (Fig. 3). For all exchangeable cations, except Ca, there was a negative relationship between RY and the concentrations of the exchangeable cations. For Ca, there was a positive relationship between exchangeable Ca concentration and RY (i.e. the greater the exchangeable concentration of Ca in the soil, the greater RY). There were significant differences between sites in the parameter coefficients of the relationships between RY and the concentrations of exchangeable cations for all exchangeable cations (Table 4). The EC50 values for the relationships between RY and exchangeable concentration of Ca and Mn were significantly (*P* < 0.05) greater at Rothamsted than at Woburn. In contrast, the EC50 values for the relationships between RY exchangeable Al was significantly (*P* < 0.05) greater at Woburn than at Rothamsted (Table 4).

**Table 4:** Log-logistic (Eq. 1) parameter coefficients for the relationship between relative yield (RY) of spring barley and soil exchangeable cations (Mn, Ca, Al) for nine years (measured cation concentrations in 1967 and estimated concentrations for 1965, 1966, 1970, 1971, 1972, 1973, 1978, 1985) at Rothamsted and Woburn

Cation	Site	b (Slope)	c (Lower Limit)	d (Upper Limit)	e (EC50^a^)
Mn	Rothamsted	9.92 (2.0)	− 0.17 (0.13)	0.98 (0.02)	451.6 (13.1)
Mn	Woburn	12.39 (2.7)	− 0.03 (0.09)	1.01 (0.01)	74.6 (1.3)
Ca	Rothamsted	− 7.50 (1.1)	− 0.06 (0.06)	1.00 (0.02)	1002.6 (25.3)
Ca	Woburn	− 9.61 (1.4)	0.00 (0.05)	1.02 (0.01)	695.8 (12.1)
Al	Rothamsted	1.29 (0.23)	− 0.08 (0.08)	0.98 (0.02)	10.8 (1.74)
Al	Woburn	4.23 (0.85)	− 0.02 (0.05)	1.01 (0.01)	26.0 (1.06)

The estimates are based upon the equations given in Table S2

Standard error of the mean (where n = 288) is given in brackets

^a^The effective concentration, i.e. cation concentration at 50% RY

At Rothamsted the concentration of exchangeable Mn was between 0 and 572 mg kg^−1^, while at Woburn Mn concentrations ranged from 0 and 100 mg kg^−1^ (Fig. 3). The log-logistic function (Eq. 1) was fitted to the relationship between RY and the concentration of exchangeable Mn (Fig. 3) and a significant difference (*P* < 0.05) was detected between the sites (Table 4). The EC50 for Mn at Rothamsted was 452 mg kg^−1^, significantly greater than the EC50 of 75 mg kg^−1^ at Woburn (Table 4).

Rothamsted had a very wide range of exchangeable Ca concentrations reaching 2500 mg kg^−1^ (Fig. 3). At Woburn the Ca concentrations were within a smaller range with few values > 1500 mg kg^−1^ (Fig. 3). At both sites RY was ≥ 1.0 at Ca concentrations between 700 and > 2500 mg kg^−1^, although plots with a RY of zero were also observed within this range. The relationship between RY and the concentration of exchangeable Ca was positive (Fig. 3) and there was a significant difference (*P* < 0.05) in the relationship between sites (Table 4). The mean EC50 for Ca at Rothamsted was 1000 mg kg^−1^, while at Woburn it was 695 mg kg^−1^.

The concentration of exchangeable Al at Rothamsted was up to 176 mg kg^−1^ which was a greater range than at Woburn with exchangeable Al concentrations < 120 mg kg^−1^ (Fig. 3). At both sites the highest concentrations were associated with zero RY, which indicates a failed crop (Fig. 3). The relationship between RY and the concentration of exchangeable Al was negative (Fig. 3) and there was a significant effect in the relationship between sites (Table 4). The EC50 for Al at Rothamsted was 10.8 mg kg^−1^, significantly less than the EC50 of 26 mg kg^−1^ at Woburn (Table 4).

### Concentrations of exchangeable cations (a) when yield is maximal and (b) when yield is reduced by soil acidity

The relationships between RY and the concentrations of soil exchangeable cations (Fig. 3) indicate a wide range of exchangeable cation concentrations where crop yield was not limited (where RY ≥ 1) and where crop yield was restricted (RY < 0.5). The concentrations of soil exchangeable cations that did not limit the yield of barley or were associated with RY < 0.5 are given for each site in Table 5.

**Table 5:** Soil exchangeable cations concentrations^a^ (mg kg^−1^) observed at Rothamsted and Woburn where: (a) relative yield was maximal and (b) relative yield was < 0.5 maximal

Cation	(a) Concentrations (mg kg^−1^) where yield was not limited Rothamsted	Woburn	(b) Concentrations (mg kg^−1^) associated with reduced yield Rothamsted	Woburn
Mn	0–417	0–74	> 417–572	> 74
Ca	1040–2500	690–900	< 1040	< 690
Al	0–7.5	0–25.7	> 7.5	> 25.7

^a^Concentrations taken from the RY-cation relationships in Fig. 3

## Discussion

### Site effects on the concentrations of exchangeable cations in the soil

This experiment was undertaken at two sites with distinctly different soil properties (Table S1). A significant site effect was detected on soil pH and on the concentration of most exchangeable cations in the soil (i.e. all except for Al) (Table 2). The Rothamsted soil was formed on plateau drift and clay with flints, while the soil at Woburn is formed on colluvium over unconsolidated sandstone (Lower Greensand). Further description of the soil parent material at each site is available (Avery and Catt 1995; Catt et al. 1980) and there is sufficient evidence to indicate that there has been contrasting pedological development at each site. Evaluation of long-term management effects on clay mineralogy at Rothamsted indicate that the greatest changes have been observed on the most acidic soil (control lime plots) (Tye et al. 2009). The limited clay mineralogy of the two soils, presented in Goulding and Loveland (1986; Table 1) shows that both soils contain mica and ‘Expansible’ clay minerals (interstratified mica-smectite), but with the latter dominant in the Woburn soil. However, the fundamental contrast between the soils is texture. The Rothamsted soil has greater clay and silt content and the Woburn soil is sandier (Table S1). This difference in texture corresponds with the greater cation exchange capacity of the Rothamsted soil compared to the Woburn soil, despite the dominance of ‘Expansible’ minerals in the latter. Both sites were subject to the same liming treatments which provided a large range in exchangeable cation concentrations. Soils with a larger ECEC have a stronger buffering ability and are comparatively less prone to leaching (Rowell 1994). The fundamental differences between the soils described above probably account for the observation that the Rothamsted soil had significantly greater concentrations of most of the exchangeable cations (i.e. Mn, Ca, Cd, Cr, Cu, Co, Zn, Mg and K) than the Woburn soil (Fig. 2). In contrast, the Woburn soil had greater concentrations of exchangeable Al and Fe than the Rothamsted soil (Fig. 2). Site differences in exchangeable cation concentrations are also influenced by the exact composition of cations, which influences whether divalent cations such as Mg^2+^ or Ca^2+^ are displaced by trivalent cations such as Al^3+^ or Fe^3+^ (White 2013). Differences in all these soil properties explain why there was a soil-type specific equation to describe the relationship between the soil pH and the concentrations of exchangeable cations in the soil (Table S2). However, according to the hypothesis proposed in the Introduction, if there is a unique concentration of a single exchangeable cation in all soils that results in toxicity to crops, then the EC50 value would be the same for both the Rothamsted and Woburn soils.

### The impact of soil type on the relationship between RY and the concentrations of exchangeable cations in the soil

There was a significant site effect on the relationship between RY and the concentrations of exchangeable Mn, Ca and Al (Fig. 3, Table 4). Several studies have indicated that the critical concentrations of soil exchangeable cations resulting in toxicity to crops can differ between soil types. For example, the critical concentrations of exchangeable Al differ between soil types (Abdulaha-Al Baquy et al. 2018; Adams 1984). These differences are presumably related to inherent differences in soil properties. Such variability in critical concentrations of exchangeable cations between soil types presents a major challenge in the development of a diagnostic approach to identify potential cation toxicities in agricultural soils. Another confounding influence on the development of diagnostic values for cation toxicities in soil is the soil extraction method, which should reflect the cation concentrations available to crops.

### Evaluation of soil extraction method in the measurement of exchangeable cations

The concentration of an exchangeable cation in soil indicates the amount of that cation held per mass of soil available to a plant (White 2013). The agronomic utility of a soil extraction method is determined by how well it estimates phytoavailable cation concentrations and relates to crop growth parameters, such as biomass or yield. A variety of different soil chemical extractions have been used to determine the concentration of a nutrient in the soil. Much research on contamination of soils has been undertaken using the total concentrations of elements (Warne et al. 2008; White et al. 2012), but total concentrations are poor predictors of potential toxicity to crops (Smolders et al. 2009).

This study used the NH_4_NO_3_ extraction method which is chemically less reactive than other methods and appears suitable for determining the concentrations of most exchangeable cations in soils (Schöning and Brümmer 2008). The NH_4_NO_3_ extraction method compares favourably to other extractable methods used to determine the phytoavailability of a wide range of cations (Mn, Fe, Ca, Mg, K, B, Zn, Mo, Ni, Cu, Cd, Pb, As, Se, Co) (Abedin et al. 2012). Indeed, Pueyo et al. (2004) observed that there was < 10% difference in estimated exchangeable cation concentrations using three common extraction methods (CaCl_2_, NaNO_3_, NH_4_NO_3_). It is therefore reasonable to assume that measuring exchangeable cations using the NH_4_NO_3_ extraction method gives an adequate estimate of the phytoavailability of cations in a soil that may be influence crop yield. Nevertheless, care must be taken when comparing the EC50 cation concentrations found in our study with the critical exchangeable cation concentrations obtained in studies using alternative methods of soil extraction (Table 1).

### Assessment of published critical concentrations for exchangeable cations in the soil

Aluminium and Mn toxicity has been reported to limit crop yields in strongly acidic soils (Conyers et al. 1991). Previous studies have reported critical concentrations of soil exchangeable cations above which the biomass or yield of barley is reduced (Table 1). These critical concentrations of exchangeable cations can be compared with the observed EC50 values observed in this study (Fig. 3; Table 5). This comparison can serve to identify the cations that are likely to become toxic to barley as the soil is acidified.

Critical concentrations of exchangeable Al < 5 mg kg^−1^ have been reported to impact barley yield (Anderson and Bell 2019) and other estimates range up to 25 mg kg^−1^ (Dolling et al. 1991). The EC50 values for Al reported here were 10.8 mg kg^−1^ at Rothamsted and 26.0 mg kg^−1^ at Woburn (Table 4). There are two important differences between these published values and the EC50 values reported in this study: (*i*) the extraction methods differ, which accounts for at least 10% of the difference, and (*ii*) the critical concentration is given for 90% RY in the published studies. It is possible that the concentrations of exchangeable Al limited barley yields at both Rothamsted and Woburn. Differences in the EC50 between Rothamsted and Woburn are likely to reflect soil properties, which influence the solubility of Al and its toxic to plants (Foy 1984). Slattery and Coventry (1993) reported that the critical Al concentration for toxicity was greater soils with a high buffering capacity and high organic carbon content. Moreover, the mineral source of Al and the quantity of organic matter control the solubility of Al (Conyers 1990) and it is Al solubility which determines the toxicity of Al to crops. The importance of Al in the soil was confirmed in the simulation of soil acidification of the Rothamsted soil, in specific for the control (no lime) treatment pH < 4.5 (Xu et al. (2020).

The published critical soil concentrations of exchangeable Mn range from 10 to 52 mg kg^−1^ (Table 1). These concentrations are much lower than the EC50 value determined at Rothamsted, and slightly lower than the EC50 value determined at Woburn (Fig. 3; Table 4). Indeed, crop yield at Rothamsted was not limited by up to 420 mg Mn kg^−1^ (Table 5), which is an order of magnitude greater than the published critical concentrations (Table 1). It is, therefore, possible that barley is not as sensitive to soil exchangeable Mn concentration as previously thought, especially at Rothamsted (Fig. 3). Differences in the critical exchangeable Mn concentration between soils could result from a variety of soil factors, including aeration and microbial activity, both of which influence the speciation of Mn and, hence, its toxicity to plants (Foy, 1984). In addition, the solubility of Mn is controlled by soil pH and the kinetics of redox reactions (Hernandez-Soriano et al. 2012). Mn oxidation and reduction is controlled by soil water potential and temperature (Sparrow and Uren 2014). Therefore, given the different basic soil properties (i.e. texture) between the experimental sites (Table S1) it is little surprise there is such a large difference in the Mn concentration which reduces barley grain yield (Table 5).

Hazelton and Murphy (2007) reported that at exchangeable Ca concentrations < 400 mg kg^−1^ in the soil Ca deficiency is possible. Thus, it is possible that the soil exchangeable Ca concentration limited barley yield at Woburn when the exchangeable Ca concentration was < 600 mg kg^−1^ and RY < 0.5, although this is unlikely to be the case for the larger exchangeable Ca concentrations in the Rothamsted soil (Fig. 3, Table 5).

The EC50 values for the relationships between RY and the concentration of exchangeable cations in the soil (Fig. 3; Table 5), together with previous estimates of the critical soil exchangeable cation concentrations (Table 1) suggests that toxic Al and Mn concentrations might limit barley yields at both Rothamsted and Woburn.

Further validation is required to determine critical soil concentrations. However the relationship between a crop response (barley RY) and key yield-limiting soil properties (i.e. exchangeable Al and Mn; Fig. 3) clearly demonstrate the importance of soil acidity on crop production. At low soil pH there is an increased solubility and thus, decreased adsorption of Al and Mn (Holland et al. 2018). In this study exchangeable cations were measured and these are a convenient indicator to assess the impact of Al and Mn on plant growth. The evidence (Table 5; Fig. 3) from this study that was used to test the hypothesis (that yield was restricted by the concentration of a single exchangeable cation in the soil) was not conclusive overall. As indicated above additional validation work is required to identify the cation which is most restricting yield. Further measurements include collecting plant tissue and root samples; root measurements have been shown to strongly relate to soil Al concentration (Valle et al. 2009). Previous studies have reported that toxicity can result from additive or synergistic interactions between cations together. This is termed “mixture toxicity”. For example, the concentrations of Zn and Cu was toxic on the shoot growth of barley (Hamels et al. 2014). The results (Table 5; Fig. 3) in this study indicate that the combined concentration of Al and Mn restricted the yield of barley at both sites. Conyers et al. (1991) found there is a complex interaction between Al and Mn in which biochemical processes regulate the plant requirement for Mn and thus the importance of Mn for plant nutrition ought not to be neglected.

### The prediction of relative yield (RY) from soil pH and three exchangeable cations (Al, Mn, Ca) at Rothamsted and Woburn

The analyses described above evaluated the relationships between RY and the concentrations of each cation separately (Fig. 3). In addition to this approach, an additive linear model can be used to predict the effect on RY from different variables by modelling key soil response variables (i.e. soil pH, Al, Mn and Ca) at Rothamsted and Woburn (Table S4). For these soil variables the R^2^ was similar at each site, being 0.60 at Rothamsted and 0.67 at Woburn. At each site the effect of the concentration of exchangeable Al was highly significant (*P* < 0.001) and had the strongest effect of any of the selected soil variables. In comparison, soil pH and the concentrations of exchangeable Mn and exchangeable Ca had much less effect on RY. Nevertheless, there were large differences between the sites in the significance (*P* values) of the effects of the concentration of exchangeable Mn and Ca which was likely due to basic soil properties (Table S1). Thus, while the effect of the concentration of exchangeable Mn was significant (*P* < 0.05) on the sandier soil at Woburn, it had no significant effect on the RY at Rothamsted.

## Conclusion

The long-term liming experiment at Rothamsted and Woburn continues to provide insights of significance for soil-crop nutrient relations that have implications for agronomy. The effect of liming was most evident in the relationship between soil pH and the concentrations of exchangeable cations, and so on the availability of potentially toxic elements to crops in the soils. Comparison of barley yields with the concentrations of exchangeable cations indicated that Al and Mn were most important in limiting crop yield. Liming also strongly influenced the concentrations of other exchangeable cations (Ca, Cd, Cr, Fe, Cu, Co, Zn and Ni), but these did not significantly reduce the yield of barley. Fundamental soil properties such as texture play an important role in controlling how liming affects the concentrations of toxic cations such as Al and Mn. Soil types which are sandier (such as the sandy loam at Woburn) are more sensitive to Mn, reducing yield compared to the silty clay loam at Rothamsted. Future research is required to (i) better understand the impacts of soil acidification and liming on crop yield, specifically to resolve the nature of multiple negative soil cation effects such as Al and Mn; (ii) confirm the observed inferences of Al and Mn on yield and to validate the critical exchangeable Al and Mn concentrations for a wide range of soils.

## Supplementary Information

Below is the link to the electronic supplementary material.

Supplementary material 1 (DOCX 24 kb)
