# Supplementary material for: Liming impacts barley yield over a wide concentration range of soil exchangeable cations
Source: Nutr Cycl Agroecosyst. 2021 Apr 25;120(2):131–44. doi: 10.1007/s10705-020-10117-2 (PMC8550446; doi:10.1007/s10705-020-10117-2)
Supplement: Supplementary file 1 — Supplementary material 1 (DOCX 24 kb) [file 10705_2020_10117_MOESM1_ESM.docx]

**Supplementary Material**

**Title**

Liming impacts barley yield over a wide concentration range of soil exchangeable cations

**Authors**

J.E. Holland^1^*, P.J. White^2^, J-N. Thauvin^2^, L. Jordan-Meille^3^, S. M. Haefele^4^, C.L. Thomas^4^, K.W.T. Goulding^4^, S.P. McGrath^4^

^1^ 1 Rhynd Farm Cottages, Leuchars, St Andrews, KY16 0DR, UK

^2^ James Hutton Institute, Dundee DD2 5DA, UK

^3^ Unité Mixte de Recherche 1391 ISPA, Bordeaux Sciences Agro, INRAE, 33140 Villenave d’Ornon, France

^4^ Department of Sustainable Agriculture Sciences, Rothamsted Research, Harpenden, Hertfordshire, AL5 2JQ, UK

* Corresponding author: J.E. Holland

**Table S1. Selected soil description and soil properties (depth 0 – 20 cm) at the Rothamsted and Woburn experimental sites**

| **Soil description/ property** | **Rothamsted** | **Woburn** |
| --- | --- | --- |
| WRB order^a^ | Luvisol | Arenosol |
| USDA Soil Taxonomy - order^b^ | Alfisol | Alfisol, Entisol, Ultisol |
| Soil series^c^ | Batcombe | Cottenham/ Stackyard |
| Texture | silty clay loam | sandy loam |
| Clay^d^ (%) (<2µm) | 20 | 12 |
| Silt^d^ (%) (2-60µm) | 52 | 17 |
| Sand^d^ (%) (60µm – 2mm) | 28 | 71 |
| Total soil carbon^e^ (%) | 1.40 | 0.82 |
| ECEC^f^ (meq 100 g^-1^) | 9.75 | 7.11 |

^a^Soil classification according to WRB ([2006](#_ENREF_43))

^b^Soil classification according to USDA Soil Taxonomy (Soil Survey Staff, 1999)

^c^Soil classification according to Avery (1980)

^d^Rothamsted from Avery & Catt (1995), profile 3; Woburn from Catt *et al* (1980), profile 1

^e^Bolton (1977)

^f^ECEC = effective cation exchange capacity

**Table S2. Regression equations (including parameter coefficients) and statistics for the relationships between soil pH and exchangeable cation concentrations (Mn, Ca, Cd, Cr, Al, Fe, Cu, Co, Zn, Ni) in the soil based on measured values from 1964, 1967, 1974, 1979, 1983 and 1989 at Rothamsted and Woburn**

| **Cation** | **Site** | **P value** | **R^2^** | **Equation** |
| --- | --- | --- | --- | --- |
| Mn | Rothamsted | <0.001 | 0.88 | Mn = 1141.5 + (148.76 🞨 pH) |
| Ca | Rothamsted | <0.001 | 0.87 | Ca = -1559.8 + (519.2 🞨 pH) |
| Cd | Rothamsted | <0.001 | 0.23 | Cd = 0.5049 + (-0.0669 🞨 pH) |
| Cr | Rothamsted | <0.001 | 0.82 | Cr = 0.2827 + (-0.0374 🞨 pH) |
| Al | Rothamsted | <0.001 | 0.84 | Al = -0.1347 + 73933062.6 🞨 exp(-3.2009 🞨 pH) |
| Fe | Rothamsted | <0.001 | 0.57 | Fe = 0.217 + 497.69 🞨 exp(-1.5872 🞨 pH)) |
| Cu | Rothamsted | <0.001 | 0.41 | Cu = 0.1003 + 11365.12 🞨 exp(-2.49 🞨 pH)) |
| Co | Rothamsted | <0.001 | 0.89 | Co = 1039.8 🞨 exp(-1.368 🞨 pH) |
| Zn | Rothamsted | <0.001 | 0.88 | Zn = 3719 🞨 exp(-1.62 🞨 pH) |
| Ni | Rothamsted | <0.001 | 0.92 | Ni = 132.9 🞨 exp(-1.20 🞨pH) |
| Mn | Woburn | <0.001 | 0.91 | Mn = 199.8 + (-25.8 🞨 pH) |
| Ca | Woburn | <0.001 | 0.88 | Ca = -1399.9 + (412.3 🞨 pH) |
| Cd | Woburn | <0.001 | 0.99 | Cd = 0.1067 + (-0.0146 🞨 pH) |
| Cr | Woburn | <0.001 | 0.67 | Cr = 0.0527 + (-0.0061 🞨 pH) |
| Al | Woburn | <0.001 | 0.81 | Al = 65691.5 🞨 exp(-1.549 🞨 pH) |
| Fe | Woburn | <0.001 | 0.65 | Fe = 0.258 + 3284.61 🞨 exp(-1.699 🞨 pH) |
| Cu | Woburn | 0.02 | 0.11 | Cu = 0.063 + 150.55 🞨 exp(-1.735 🞨 pH) |
| Co | Woburn | <0.001 | 0.87 | Co = 57.321 🞨 exp(-1.02 🞨 pH) |
| Zn | Woburn | <0.001 | 0.94 | Zn = 3.175 + (-0.4396 🞨 pH) |
| Ni | Woburn | <0.001 | 0.89 | Ni = 129.5 🞨 exp(-1.205 🞨 pH) |

**Table S3. The Pearson correlation coefficient (r value) between the exchangeable cations (Mn, Ca, Cd, Cr, Al, Fe, Cu, Co, Zn, Ni, Mg, K) in the soil based on measured concentrations in 1967 and estimated^a^ concentrations for 1965, 1966, 1970, 1971, 1972, 1973, 1978, 1985 at Rothamsted and Woburn**

| **Site** | **Cation** | **Ca** | **Cd** | **Cr** | **Al** | **Fe** | **Cu** | **Co** | **Zn** | **Ni** |
| --- | --- | --- | --- | --- | --- | --- | --- | --- | --- | --- |
| Rothamsted | Mn | -1.00 | 0.87 | 0.98 | 0.60 | 0.81 | 0.65 | 0.86 | 0.81 | 0.88 |
|  | Ca |  | -0.85 | -0.98 | -0.60 | -0.81 | -0.64 | -0.85 | -0.80 | -0.88 |
|  | Cd |  |  | 0.86 | 0.48 | 0.71 | 0.56 | 0.74 | 0.72 | 0.79 |
|  | Cr |  |  |  | 0.60 | 0.81 | 0.65 | 0.85 | 0.81 | 0.88 |
|  | Al |  |  |  |  | 0.91 | 0.94 | 0.89 | 0.89 | 0.86 |
|  | Fe |  |  |  |  |  | 0.93 | 0.99 | 0.97 | 0.98 |
|  | Cu |  |  |  |  |  |  | 0.92 | 0.96 | 0.90 |
|  | Co |  |  |  |  |  |  |  | 0.98 | 1.00 |
|  | Zn |  |  |  |  |  |  |  |  | 0.98 |
| Woburn | Mn | -0.99 | 0.83 | 0.97 | 0.81 | 0.79 | 0.49 | 0.91 | 0.98 | 0.88 |
|  | Ca |  | -0.80 | -0.95 | -0.81 | -0.79 | -0.46 | -0.90 | -0.96 | -0.87 |
|  | Cd |  |  | 0.89 | 0.71 | 0.75 | 0.57 | 0.83 | 0.91 | 0.80 |
|  | Cr |  |  |  | 0.78 | 0.79 | 0.59 | 0.90 | 0.99 | 0.87 |
|  | Al |  |  |  |  | 0.99 | 0.65 | 0.97 | 0.81 | 0.98 |
|  | Fe |  |  |  |  |  | 0.69 | 0.97 | 0.82 | 0.98 |
|  | Cu |  |  |  |  |  |  | 0.64 | 0.56 | 0.67 |
|  | Co |  |  |  |  |  |  |  | 0.92 | 1.00 |
|  | Zn |  |  |  |  |  |  |  |  | 0.89 |

^a^ The estimates are based upon the equations given in Table S2

**Table S4. The prediction of relative yield (RY) at each site (Rothamsted and Woburn) with key soil variables using an additive linear model^a^**

| **Site** | **Response variable** | **P value** |
| --- | --- | --- |
| Rothamsted | pH | 0.064 |
| Rothamsted | Mn | 0.070 |
| Rothamsted | Ca | 0.073 |
| Rothamsted | Al | <0.001 |
| Woburn | pH | 0.087 |
| Woburn | Mn | 0.032 |
| Woburn | Ca | 0.788 |
| Woburn | Al | <0.001 |

^a^ RYi = ∑kj.variable ij + εi; where RYi= Relative yield of the plot i, kj = regression coefficient for the variable j, variable ij= soils exchangeable cations concentration or soil pH, for cation j and plot i, εi = residual
